# Impact of abnormal metabolic-immunoinflammatory pathway on splenomegaly in patients with chronic schizophrenia and exploration of risk factors: case-control study

**DOI:** 10.3389/fpsyt.2026.1790346

**Published:** 2026-04-02

**Authors:** Yang Shen, Di Wang, Zhiyong Li

**Affiliations:** 1 Department of Clinical Five, Beijing HuiLongGuan Hospital, Capital Medical School, Peking University HuiLongGuan Clinical Medical School, Beijing, China; 2 Department of Psychiatry, Chuiyangliu Hospital affiliated with Tsinghua University, Beijing, China; 3 Department of Psychiatry, Beijing Changping Hospital of Integrated Chinese and Western Medicine, Beijing, China

**Keywords:** abnormalities, aripiprazole, chronic, immunoinflammation, metabolic, schizophrenia, splenomegaly

## Abstract

**Objective:**

This study aimed to identify risk factors for splenomegaly in chronic schizophrenia patients and clarify associations among metabolic−immunoinflammatory pathways, psychiatric symptoms and splenomegaly. The findings will help optimize somatic monitoring and intervention strategies.

**Methods:**

A case−control design was used. A total of 426 patients were assigned to splenomegaly (n= 165) and non−splenomegaly (n= 261) groups according to abdominal ultrasound. Demographic data, clinical information, and antipsychotic use were collected. Mental symptoms were assessed by the Positive and Negative Syndrome Scale. Hematological indicators were detected, and abdominal ultrasound was performed to evaluate spleen morphology and fatty liver occurrence. SPSS 24.0 was used for statistical analysis, including univariate analysis and binary logistic regression to screen influencing factors of splenomegaly.

**Results:**

The splenomegaly group had significantly higher levels of lipoprotein(a), cholesterol, triglycerides, HbA1c, CRP, IL-6 and β2-microglobulin than the non-splenomegaly group (all *p* < 0.05). The incidence of fatty liver and PANSS negative symptom score were significantly higher in the splenomegaly group, while the usage rate of aripiprazole was lower (*p< 0.05*). Binary logistic regression showed that HbA1c (OR = 1.797, *p* = 0.046) and PANSS negative symptom score (OR = 2.258, *p* = 0.003) were independently associated with splenomegaly. Aripiprazole use was associated with lower odds of splenomegaly (OR = 0.656, *p* = 0.041).

**Conclusion:**

Splenomegaly in chronic schizophrenia patients is closely linked to metabolic abnormalities and immunoinflammatory activation. Prominent negative symptoms are independently associated with splenomegaly and may serve as an early warning signal. Aripiprazole use is independently associated with reduced odds of splenomegaly.

## 1 Introduction

Schizophrenia is a chronic, progressive severe mental disorder. Its global prevalence remains around 1%, and over 60% of patients are in the chronic phase (1, 2). It is a chronic, relapsing disorder requiring long−term or lifelong antipsychotic treatment (3). Although the survival of patients with schizophrenia has prolonged with improved diagnosis and treatment. However, the adverse effects caused by long-term use of antipsychotic drugs are also quite prominent. Somatic comorbidities have become a major cause of reduced quality of life and poor prognosis, and have received attention in current research (4, 5).

Some studies have shown that the incidence of somatic diseases such as metabolic syndrome, liver injury, and immune dysfunction in chronic schizophrenia patients is 2–3 times higher than that in the general population (6). Most patients have impaired ability to perceive and report physical discomfort due to psychiatric symptoms, resulting in delayed detection and intervention of somatic complications (7, 8). Therefore, exploring the potential mechanisms of somatic organ damage in chronic schizophrenia patients, identifying high-risk factors, and establishing targeted monitoring and intervention systems have become important clinical issues in psychiatry.

Numerous studies show that patients with chronic schizophrenia commonly have metabolic disorders, including dyslipidemia, impaired glucose regulation and a high prevalence of fatty liver (9). Abnormal metabolism may be correlated with the imbalance of immune function. A variety of factors can affect the immune function status of patients, including long-term use of antipsychotics, pathophysiological changes of the disease itself, and unhealthy lifestyles (10, 11). According to the immunological inflammation hypothesis of schizophrenia, patients exhibit chronic low-level inflammation in both the peripheral nervous system and the central nervous system. This immune alteration causes insulin resistance, dysglycemia, dyslipidemia, obesity and other metabolic disorders. At the same time, these metabolic disorders can further amplify the inflammatory response and participate in the occurrence of mental symptoms and the development of metabolic complications (12).

A large number of studies have confirmed that the levels of pro-inflammatory factors, such as C-reactive protein (CRP) and interleukin-6 (IL-6), are significantly elevated in the peripheral blood of patients with schizophrenia. This indicates the existence of an abnormal immune state in these patients (13). However, whether the structure and function of the spleen, as a core regulatory organ of inflammatory response, are affected by this state has not been fully clarified. As a key immune organ and metabolic regulator, the spleen may contribute to somatic impairment in schizophrenia via immune activation and inflammation, such as liver disorders, hematological abnormalities, and metabolic disorders (14, 15). The current studies mainly focus on these common physical problems, but do not pay sufficient attention to the changes in the spleen (16).

Studying the morphology and function of the spleen in patients with chronic schizophrenia holds significant clinical importance. Splenomegaly may not only further aggravate immune dysfunction, but also serve as a “visual marker” of abnormal metabolic-inflammatory pathways, providing clues for identification of somatic damage (17). Chronic schizophrenia patients often have imbalanced diet and exercise due to negative symptoms. Long-term use of antipsychotics may also affect metabolism and immune function (18). These factors together constitute potential causes of splenomegaly, which in turn may affect the physical health of patients, forming a vicious cycle of “mental symptoms-somatic abnormalities-aggravated symptoms” (19). Clinical evaluation in schizophrenia focuses mainly on psychiatric symptoms, and routine monitoring of peripheral organ morphology is inadequate. Some patients are found to have splenomegaly only when accompanied by obvious metabolic or immune damage, missing the opportunity for early intervention (20).

Furthermore, the common adverse reactions of most second-generation antipsychotic drugs are related to metabolic issues. During the course of drug treatment, this may continue to affect the morphology and function of the spleen. Aripiprazole, as a new generation of medication, exhibits a favorable metabolic and inflammatory profile compared with olanzapine, clozapine, and other high-metabolic-risk antipsychotics (21). Emerging evidence indicates that aripiprazole is associated with lower risks of dyslipidemia, glucose dysregulation, and excessive weight gain, and may exert mild anti-inflammatory effects (22). These properties suggest that aripiprazole might modulate immune-metabolic pathways and influence peripheral organ morphology. However, direct evidence linking aripiprazole use to splenomegaly in chronic schizophrenia is still lacking.

Therefore, Systematic investigation of the prevalence, risk factors and mechanisms of splenomegaly in chronic schizophrenia can help optimize somatic monitoring and personalized interventions, which is of great for improving the prognosis of patients. This study focused on patients with chronic schizophrenia, aiming to explore the influencing factors of splenomegaly and the effects of antipsychotic drugs. We analyzed the possible connections among “metabolism-inflammation-psychological symptoms-splenomegaly”, providing a new research perspective for clarifying the pathophysiological mechanism of splenomegaly.

## 2 Methodology

### 2.1 Study design and participants

This study adopted a case-control design. Chronic schizophrenia patients admitted to Beijing Changping Hospital of Integrated Traditional Chinese and Western Medicine were selected as the research objects. The study protocol was approved by the Ethics Committee of Beijing Changping Hospital of Integrated Traditional Chinese and Western Medicine. All included patients signed informed consent forms. Inclusion criteria: (1) Meeting the diagnostic criteria for schizophrenia in the 10th Revision of *the International Classification of Diseases* (ICD-10) (23), with a disease course ≥5 years, diagnosed as chronic schizophrenia; (2) Aged 18–65 years, with clear consciousness, able to cooperate with scale assessment, physical examinations, and laboratory tests; (3) Receiving long-term antipsychotic treatment for ≥1 year, with stable medication regimen for ≥3 months. Exclusion criteria: (1) Complicating with somatic diseases that may cause splenomegaly, such as cirrhosis, portal hypertension, hematological diseases, infectious diseases, and autoimmune diseases; (2) Complicating with organic brain diseases or other mental disorders, such as bipolar disorder, substance dependence, and intellectual disability; (3) Having a history of major surgery, severe infection, or drug abuse within 3 months; (4) The patient’s body mass index is less than 30kg/M^2^, No history of diabetes or blood glucose has remained stable for more than half a year; (5) Incomplete clinical data, unable to complete subsequent statistical analysis.

According to the results of abdominal ultrasound, the included patients were divided into the splenomegaly group and non-splenomegaly group. The diagnostic criteria for splenomegaly referred to previous studies (24): spleen thickness > 40 mm, or spleen length > 120 mm, or spleen volume > 400 cm³. A total of 426 patients were included, including 165 in the splenomegaly group and 261 in the non-splenomegaly group.

### 2.2 Study tools and assessment methods

#### 2.2.1 Collection of demographic and clinical data

A self-designed general data questionnaire was used to collect patients’ demographic information, including age, gender, educational level, disease course, height, weight, blood pressure, and specific type, dosage, and drug class of antipsychotics, as well as use of adjunctive medications. Body mass index (BMI) was calculated based on height and weight. Antipsychotic doses were converted into chlorpromazine equivalent doses according to the specifications in *Psychiatric Pharmacotherapy (*25). Single-drug doses were converted directly, and combined-drug doses were the sum of equivalent doses of each drug.

Antipsychotics were categorized into two classes: (1) second-generation antipsychotics with higher metabolic risk (olanzapine, clozapine, quetiapine); (2) second-generation antipsychotics with lower metabolic risk (aripiprazole, risperidone).

#### 2.2.2 Mental symptom assessment tool

The Positive and Negative Syndrome Scale (PANSS) was used to assess the severity of patients’ mental symptoms (26). The scale consists of 30 items, divided into the Positive Symptom Scale (P scale, 7 items), Negative Symptom Scale (N scale, 7 items), and General Psychopathology Scale (G scale, 16 items). Assessments were completed by two trained attending psychiatrists. Inter-rater reliability test was performed before assessment, and the results showed that the intraclass correlation coefficient (ICC) > 0.85, indicating good inter-rater consistency and ensuring the reliability of assessment results.

#### 2.2.3 Abdominal ultrasound examination

All enrolled patients underwent abdominal ultrasound as a standardized routine somatic health monitoring examination for chronic schizophrenia in our hospital, rather than being performed due to clinical symptoms, suspected splenic abnormalities, or other targeted indications. For long-term hospitalized patients, abdominal ultrasound is rechecked every 3–9 months to timely identify pathological changes in important abdominal organs such as fatty liver.

A color Doppler ultrasound diagnostic instrument (model: GE E95) with a probe frequency of 3.5~5.0 MHz was used. Patients fasted for more than 8 hours before the examination, taking supine and left lateral positions. Routine scanning of the spleen was performed to measure its thickness, length, and volume, and to determine the presence of splenomegaly. Meanwhile, liver morphology and echo were observed to assess the occurrence of fatty liver. The diagnostic criteria for fatty liver referred to the Guidelines for the Diagnosis and Treatment of Non-Alcoholic Fatty Liver Disease (27). All ultrasound examinations were completed by a physician with more than 5 years of experience in abdominal ultrasound diagnosis and treatment, and the operation strictly followed ultrasound examination specifications.

#### 2.2.4 Hematological index detection

Sample collection: In the early morning fasting state, professional nurses collected 5~8 mL of elbow venous blood from patients and placed it in vacuum blood collection tubes. Among them, 2 mL was used for glycated hemoglobin (HbA1c) detection. The remaining blood samples were allowed to stand at room temperature for 30 minutes, then centrifuged at 3000 r/min for 10 minutes to separate serum. The serum was stored in a -20°C refrigerator and detected within 24 hours.

A fully automatic biochemical analyzer (model: Beckman Coulter AU5800) was used to detect cholesterol (CHO), triglycerides (TG), fasting blood glucose, lactate dehydrogenase, apolipoprotein A1/B, high-density and low-density lipoprotein, uric acid, urea, creatinine, γ-glutamyl transferase, alkaline phosphatase, albumin, globulin, total protein, direct bilirubin, total bilirubin, aspartate aminotransferase, and alanine aminotransferase by enzymatic method. Immunoturbidimetry was used to detect lipoprotein (a) (LPa), β2-microglobulin (β2-MG), and C-reactive protein (CRP). High-performance liquid chromatography (HPLC) was used to detect HbA1c. Enzyme-linked immunosorbent assay (ELISA) was used to detect interleukin-6 (IL-6), with kits purchased from Shanghai Enzyme-Linked Biotechnology Co., Ltd. All detections strictly followed kit instructions and instrument operation specifications to ensure accurate and reliable results.

## 4 Statistical analysis

SPSS 24.0 statistical software was used for data processing and analysis. The normality of the data distribution was formally assessed using Shapiro-Wilk test. For missing data, multiple imputation was applied to ensure the reliability of the statistical results. Quantitative data were expressed as mean ± standard deviation (x ± s), and independent samples *t-test* was used for inter-group comparison. Qualitative data were expressed as frequency (n) and percentage (%), and *chi-square* test was used for inter-group comparison. Taking the occurrence of splenomegaly as the dependent variable, indicators with statistically significant differences in univariate analysis and potential confounding factors were included in binary logistic regression analysis. Stepwise regression was used to screen the influencing factors of splenomegaly. To further verify adequacy, *post hoc* power analysis was performed with a type I error rate (α) of 0.05 and a target power of 0.80. The test level α=0.05, and *p<0.05* was considered statistically significant.

## 3 Results

### 3.1 Difference analysis of demographic data

A total of 426 chronic schizophrenia patients were included in this study. The splenomegaly group had 165 cases with a mean age of 48.34 ± 11.07 years, and 50.9% were female. The non-splenomegaly group had 261 cases with a mean age of 49.31 ± 12.16 years, and 43.7% were female. There were no statistically significant differences in age and gender distribution between the two groups (*p>0.05*).

In terms of educational level, the splenomegaly group included 23 cases with primary school education or below, 72 with junior high school education, 52 with senior high school education, and 16 with college education or above. The non-splenomegaly group included 32 cases with primary school education or below, 105 with junior high school education, 76 with senior high school education, and 48 with college education or above. Statistical analysis showed no significant difference in educational level between the two groups (*p>0.05*). The total disease course was 25.61 ± 11.97 years in the splenomegaly group and 24.33 ± 11.44 years in the non-splenomegaly group, with no statistical difference(*p>0.05*), indicating that disease course was not significantly associated with splenomegaly.

In terms of physical indicators, the splenomegaly group had a height of 1.67 ± 0.07 m, weight of 67.99 ± 13.53 kg, BMI of 24.34 ± 4.62 kg/m², and systolic blood pressure of 124.61 ± 10.57 mmHg. The non-splenomegaly group had a height of 1.66 ± 0.07 m, weight of 67.70 ± 13.45 kg, BMI of 25.01 ± 4.64 kg/m², and systolic blood pressure of 124.90 ± 11.61 mmHg. There were no significant differences in these indicators between the two groups (*p>0.05*). The diastolic blood pressure in the splenomegaly group was 79.49 ± 6.52 mmHg, which was significantly higher than that in the non-splenomegaly group (78.04 ± 7.56 mmHg, *p=0.037*). Abdominal ultrasound results showed that the incidence of fatty liver was 52.1% in the splenomegaly group, significantly higher than 40.2% in the non-splenomegaly group (*p=0.016*).

Diastolic blood pressure was significantly higher in the splenomegaly group (79.49 ± 6.52 vs. 78.04 ± 7.56 mmHg, *p* = 0.037). However, the absolute between−group difference was small and within the normal physiological range, indicating statistical significance but limited clinical relevance.

Regarding drug treatment, 52 cases (31.5%) in the splenomegaly group used aripiprazole alone or in combination, compared with 112 cases (42.9%) in the non-splenomegaly group. Statistics showed that the incidence of splenomegaly was significantly lower in patients using aripiprazole than in those not using it (*p=0.019*). The remaining patients in both groups were treated with risperidone, olanzapine, quetiapine, etc., either alone or in combination of two drugs. The chlorpromazine equivalent dose was 269.63 ± 198.45 mg in the splenomegaly group and 264.48 ± 205.51 mg in the non-splenomegaly group, with no statistical difference (*p>0.05*) (See **Table 1**.)

**Table 1:** Demographic data differences analysis of the two Groups of patients.

Variables	Splenomegaly group (n=165)	Non-Splenomegaly group (n=261)	*t/χ² -value*	*p-value*
Age (years)	48.34 ± 11.07	49.31 ± 12.16	-0.848	0.397
Gender (F/M)	84/81	114/147	2.125	0.145
Total Disease Duration (months)	25.61 ± 11.97	24.33 ± 11.44	1.099	0.273
Height (m)	1.67 ± 0.07	1.66 ± 0.07	3.586	0.035
Weight (kg)	67.99 ± 13.53	67.70 ± 13.45	0.221	0.825
BMI (kg/m²)	24.34 ± 4.62	25.01 ± 4.64	-1.447	0.149
Systolic Blood Pressure (mmHg)	124.61 ± 10.57	124.90 ± 11.61	-0.264	0.792
Diastolic Blood Pressure (mmHg)	79.49 ± 6.52	78.04 ± 7.56	2.098	0.037*
Fatty Liver (n%)	52.1%	40.2%	5.780	0.016*
Chlorpromazine Equivalent Dose (mg)	269.63 ± 198.45	264.48 ± 205.51	0.255	0.799
Aripiprazole Usage Rate (n%)	31.5%	42.9%	5.546	0.019*

F, Female; M, Male. *p < 0.05 indicates statistical significance.

### 3.2 Difference analysis of mental symptoms between the two groups

PANSS results showed no statistically significant differences in PANSS total score, Positive Scale total score and item scores between the two groups (*p>0.05*). In terms of negative symptoms, the Negative Scale total score in the splenomegaly group was significantly higher than that in the non-splenomegaly group (*p=0.002*). Among them, the scores of Blunted affect, Affective Communication Disturbance, Passive-Apathetic Withdrawal, Difficulty in Abstract Thinking, and stereotyped thinking were significantly higher in the splenomegaly group (*p<0.05*). There were no significant differences in the scores of social withdrawal and lack of spontaneity and flow between the two groups (*p>0.05*).

In terms of general symptoms, the scores of Anxiety, Tension, Depression, Active social avoidance, and Work Inability scores in the splenomegaly group were significantly lower than those in the non-splenomegaly group (*p<0.05*). There were no significant differences in General Psychopathology Scale total score and other item scores between the two groups (*p>0.05*). These results suggest that patients in the splenomegaly group have more prominent negative symptoms, and the persistence of negative symptoms may be associated with somatic damage. (See **Table 2**; full data see **Supplementary Table 1**).

**Table 2:** Difference analysis of PANSS scale scores between the two groups.

Variables	Splenomegaly group (n=165)	Non-Splenomegaly group (n=261)	*t-value*	*p-value*
P Scale Total Score	15.91 ± 6.70	15.66 ± 6.09	0.397	0.692
N Scale Total Score	18.67 ± 7.82	16.48 ± 5.37	3.167	0.002*
G Scale Total Score	26.39 ± 12.40	28.81 ± 10.47	-2.658	0.471
PANSS Total Score	59.98 ± 16.86	62.94 ± 19.87	-1.649	0.100
Blunted Affect	2.95 ± 1.32	2.69 ± 1.06	2.280	0.023*
Affective Communication Disturbance	2.88 ± 1.75	2.34 ± 1.09	3.578	0.000*
Passive-Apathetic Withdrawal	3.19 ± 1.49	2.85 ± 0.98	2.588	0.010*
Difficulty in Abstract Thinking	2.39 ± 1.22	1.96 ± 1.04	3.718	0.000*
Stereotyped Thinking	2.27 ± 1.14	1.98 ± 1.03	2.703	0.007*
Anxiety	1.46 ± 1.03	1.95 ± 1.09	-4.708	0.000*
Tension	1.30 ± 0.66	1.68 ± 0.96	-4.871	0.000*
Depression	1.27 ± 0.69	1.44 ± 0.77	-2.391	0.017*
Active Social Avoidance	2.42 ± 1.46	2.85 ± 1.33	-3.056	0.002*
Work Inability	1.28 ± 0.88	1.52 ± 0.91	-2.612	0.009*

PANSS, Positive and Negative Syndrome Scale; P Scale, Positive Scale; N Scale, Negative Scale; G Scale, General Psychopathology Scale. *p < 0.05 indicates statistical significance.

### 3.3 Difference analysis of hematological test results between the two groups

Hematological analysis showed that the levels of LPa, CHO, and TG in the splenomegaly group were significantly higher than those in the non-splenomegaly group (*p<0.01*), indicating obvious dyslipidemia in the splenomegaly group. The level of HbA1c in the splenomegaly group was significantly higher than that in the non-splenomegaly group (*p=0.005*), but there was no statistical difference in fasting blood glucose between the two groups (*p=0.157*), and the mean fasting blood glucose was within the normal range. This suggests that patients in the splenomegaly group may have a long-term risk of abnormal blood glucose.

In terms of inflammatory factors and immune-related indicators, the levels of CRP, IL-6, and β2-MG in the splenomegaly group were significantly higher than those in the non-splenomegaly group (*p<0.05*), indicating more significant immune dysfunction in the splenomegaly group. Other indicators related to liver and kidney function (such as lactate dehydrogenase, apolipoprotein A1/B, high-density and low-density lipoprotein, uric acid, urea, creatinine, etc.) showed no significant differences between the two groups (*p>0.05*). (See **Table 3**.)

**Table 3:** Analysis of differences in hematological test results between the two groups of patients.

Variables	Splenomegaly group (n=165)	Non-Splenomegaly group (n=261)	*t-value*	*p-value*
Lipoprotein(a)	537.92 ± 714.88	335.76 ± 406.28	3.310	0.001*
Beta-2 Microglobulin	1.59 ± 0.56	1.47 ± 0.51	2.192	0.029*
Glucose	5.31 ± 1.42	5.60 ± 2.88	-1.417	0.157
Lactate Dehydrogenase	160.99 ± 42.93	166.43 ± 49.00	-1.205	0.229
Apolipoprotein B	0.84 ± 0.23	0.86 ± 0.35	-0.432	0.666
Apolipoprotein A1	1.16 ± 0.14	1.16 ± 0.19	-0.454	0.650
Low-Density Lipoprotein	3.25 ± 1.22	3.14 ± 1.01	0.975	0.330
High-Density Lipoprotein	1.08 ± 0.23	1.09 ± 0.27	-0.105	0.916
Cholesterol	7.14 ± 5.99	5.62 ± 4.45	2.803	0.005*
Triglyceride	3.05 ± 1.29	2.50 ± 1.12	4.584	0.000*
Uric Acid	299.71 ± 95.17	290.34 ± 105.80	0.948	0.344
Urea	4.13 ± 1.81	4.66 ± 4.94	-1.572	0.117
Creatinine	70.70 ± 23.42	67.02 ± 22.58	1.603	0.110
Gamma-Glutamyl Transferase	21.57 ± 13.03	23.65 ± 15.61	-1.481	0.139
Alkaline Phosphatase	73.60 ± 28.99	71.69 ± 23.80	0.708	0.479
Albumin	41.30 ± 3.16	41.46 ± 4.16	-0.453	0.651
Globulin	27.21 ± 3.97	26.94 ± 4.22	0.657	0.512
Total Protein	67.74 ± 6.19	67.80 ± 7.09	-0.104	0.918
Direct Bilirubin	4.76 ± 2.14	4.41 ± 1.83	1.741	0.083
Total Bilirubin	13.13 ± 6.41	13.12 ± 12.27	0.002	0.998
Aspartate Aminotransferase	20.06 ± 10.91	21.21 ± 11.38	-1.042	0.298
Alanine Aminotransferase	19.73 ± 11.83	22.15 ± 19.81	-1.581	0.115
Glycated Hemoglobin A1c	6.36 ± 1.64	5.94 ± 1.15	2.853	0.005*
C-Reactive Protein	8.85 ± 5.55	7.67 ± 5.37	2.178	0.030*
Interleukin-6	4.08 ± 2.01	3.68 ± 1.28	2.272	0.024*

*p < 0.05 indicates statistical significance.

### 3.4 Binary logistic regression analysis of influencing factors for splenomegaly

Taking the occurrence of splenomegaly as the dependent variable, indicators with statistically significant differences between the two groups and potential confounding factors were included in binary logistic regression analysis.

All variables with p< 0.05 in univariate between−group comparisons were included. These included: Age, BMI, disease duration, diastolic blood pressure, fatty liver, aripiprazole use, lipoprotein(a), total cholesterol, triglyceride, HbA1c, CRP, IL-6, β2-microglobulin, PANSS negative symptom total score. Key clinical confounders known to be associated with splenomegaly, metabolic disturbance, inflammation, and antipsychotic effects were added to control for confounding. The *post hoc* power analysis results confirmed that the sample of 426 participants provided adequate statistical power (≥80%) for the present exploratory logistic regression model.

The results showed that HbA1c level (OR = 1.797, 95%CI: 1.534-2.189, *p=0.046*) and PANSS negative symptom total score (OR = 2.258, 95%CI: 1.903-3.043, *p=0.003*) were independently associated with splenomegaly in chronic schizophrenia patients. Treatment with aripiprazole (OR = 0.656, 95%CI: 0.200-0.853, *p=0.041*) was independently associated with lower odds of splenomegaly. These findings suggest that clinical monitoring of HbA1c level and negative symptoms should be strengthened, and aripiprazole should be preferred in drug selection to reduce the risk of somatic damage in patients. (See **Table 4**.)

**Table 4:** Results of binary logistic regression analysis on factors affecting enlarged spleen.

Variables	B	Wald	*p-value*	Exp(B)	95%CI
Age	0.010	0.089	0.766	1.010	0.944-1.081
BMI	-0.921	1.945	0.163	0.398	0.109-1.452
Disease Duration	-0.048	2.375	0.123	0.954	0.898-1.013
Lipoprotein(a)	-0.001	1.443	0.030*	0.899	0.798-1.049
β2-MG	0.526	0.923	0.337	1.693	0.578-4.955
Glucose	0.341	1.178	0.278	1.407	0.759-2.606
Cholesterol	-0.282	15.912	0.000*	1.054	0.656-1.866
Triglyceride	-0.153	0.423	0.516	0.858	0.540-1.362
HbA1c	0.227	1.239	0.046*	1.797	1.534-2.189
CRP	-0.146	7.447	0.006	0.864	0.778-.960
IL-6	-0.102	0.345	0.557	0.903	0.642-1.269
N Scale Total Score	0.505	2.660	0.003*	2.258	1.903-3.043
Aripiprazole Usage Rate	-0.618	4.187	0.041*	0.656	0.200-0.853

BMI, Body mass index; β2-MG, Beta-2 Microglobulin; CRP, C-Reactive Protein; IL-6, Interleukin-6; CI, Confidence Interval. *p<0.05 indicates statistical significance.

## 4 Discussion

This study found that patients in the splenomegaly group with chronic schizophrenia had significantly higher levels of lipoprotein a LPa, CHO, and TG than those in the non-splenomegaly group. The level of HbA1c was also significantly increased, and the incidence of fatty liver was significantly higher than that in the non-splenomegaly group. These observations are consistent with previous literature reporting a high burden of metabolic disturbances in patients with schizophrenia (28, 29).

Existing evidence indicates that patients with schizophrenia have a higher prevalence of metabolic syndrome relative to the general population, related to disease pathophysiology, antipsychotic exposure, and lifestyle factors (30). Dyslipidemia and impaired glucose regulation are particularly common. Theoretically, lipid deposition in splenic vascular endothelium could trigger local inflammatory responses that might contribute to compensatory splenic enlargement (31). Elevated HbA1c reflects long-term glycemic dysregulation, which could potentially impair splenic immune cell function via oxidative stress and contribute to altered splenic morphology, even in the setting of normal fasting glucose (32). The co-occurrence of fatty liver and splenomegaly may suggest a shared metabolic-inflammatory pathophysiology; inflammatory mediators released from the steatotic liver could influence the spleen via the portal circulation, hypothetically forming a liver-spleen inflammatory axis (33, 34). These interpretations remain theoretical and require further mechanistic and longitudinal validation.

The present study also showed significantly higher levels of CRP, IL-6, and β2-microglobulin in the splenomegaly group, indicating that splenomegaly in chronic schizophrenia is associated with immune-inflammatory dysfunction (34, 35). The spleen is a central immune organ, and splenomegaly is a recognized marker of immune activation. Theoretically, pro-inflammatory cytokines such as IL-6 may stimulate lymphocyte proliferation and macrophage activation in the spleen, potentially contributing to increased splenic volume, and an enlarged spleen might in turn enhance inflammatory mediator release. However, the directionality and causal nature of this feedback loop cannot be determined from the current cross-sectional data (36). These interpretations align with the immune-inflammatory hypothesis of schizophrenia, which posits persistent low-grade inflammation in affected individuals (37). Cytokine elevations have been widely reported in schizophrenia and linked to somatic comorbidities. The present findings extend these observations by demonstrating an association between immune-inflammatory disturbances and splenic morphological abnormalities, providing supportive evidence for central-eripheral immune dysregulation in schizophrenia (38, 39).

Notably, the splenomegaly group had significantly higher PANSS negative symptom total scores, particularly for blunted affect, affective communication disturbance, passive-apathetic withdrawal, difficulty in abstract thinking, and stereotyped thinking, with no between−group differences in positive symptoms. This observation highlights a robust association between prominent negative symptoms and splenomegaly. Theoretically, negative symptoms may contribute to unhealthy lifestyle patterns that exacerbate metabolic and immune dysregulation, indirectly relating to splenic enlargement (40). Inflammatory mediators such as IL−6 may also influence dopamine and serotonin pathways, potentially worsening negative symptoms while contributing to peripheral immune organ remodeling (41). These mechanistic interpretations are speculative and require longitudinal and experimental confirmation. The present findings support the view that negative symptoms are associated with increased risk of somatic comorbidity and may serve as a clinical indicator for monitoring splenic and metabolic health (42).

In this study, aripiprazole use was associated with a lower prevalence of splenomegaly in univariate analysis, and binary logistic regression identified aripiprazole as an independently associated with reduced odds of splenomegaly (OR = 0.656, 95%CI: 0.200-0.853, *p* = 0.041), with no between-group difference in chlorpromazine-equivalent dose. Aripiprazole is an atypical antipsychotic with partial dopamine D2 agonist properties and a relatively favorable metabolic profile compared with some other antipsychotics (21, 43). The observed association does not establish a causal protective effect, and preferential use of aripiprazole to reduce splenomegaly risk requires confirmation in prospective or randomized controlled trials. The use of aripiprazole may help reduce the physical impairments associated with chronic schizophrenia (12).

Several unmeasured factors may confound the observed associations, including alcohol use, viral hepatitis status, smoking status, detailed dietary composition, and habitual physical activity. These factors were not systematically assessed in the present study and may influence metabolic, inflammatory, and splenic phenotypes. The single-center, cross-sectional design limits causal inference and generalizability. Sample size and lack of grading of splenomegaly severity also restrict interpretation. Although we recorded stable antipsychotic treatment for ≥3 months, we did not perform detailed stratification by antipsychotic metabolic risk class, specific polypharmacy patterns, or adjunctive medications. These agents may influence metabolic and immunoinflammatory outcomes. In addition, multiple testing correction was not applied in univariate analyses because they served only for exploratory variable screening, and final inferences were based on adjusted logistic regression models.

In future research, multi-center, large-sample longitudinal cohort studies can be carried out to include different regions, disease stages, detailed antipsychotic classification and concomitant treatment data. Long-term follow-up of dynamic changes in spleen morphology, metabolic-inflammatory indicators, and mental symptoms can clarify the causal relationship between variables and establish a splenomegaly risk prediction model.

## 5 Conclusion

Splenomegaly in chronic schizophrenia patients is closely associated with metabolic abnormalities and immunoinflammatory activation. Prominent negative symptoms are independently associated with splenomegaly and may serve as an early warning signal. Aripiprazole use is independently associated with reduced odds of splenomegaly.

These cross-sectional findings highlight clinically relevant associations among metabolic-immune dysregulation, negative symptoms, and splenic morphology. Future prospective and mechanistic studies are needed to clarify causal pathways and evaluate the efficacy of targeted monitoring and interventions to reduce somatic comorbidity burden in chronic schizophrenia.

## Data Availability

The original contributions presented in the study are included in the article/**Supplementary Material**. Further inquiries can be directed to the corresponding authors.
