# Supplementary material for: Impact of abnormal metabolic-immunoinflammatory pathway on splenomegaly in patients with chronic schizophrenia and exploration of risk factors: case-control study
Source: Front Psychiatry. 2026 Apr 2;17:1790346. doi: 10.3389/fpsyt.2026.1790346 (PMC13083167; doi:10.3389/fpsyt.2026.1790346)
Supplement: Supplementary file 1 [file DataSheet1.pdf]

Supplementary Table 1. Difference Analysis of PANSS Scale Scores between the Two Groups.

Variables	Splenomegaly Group (n=165)	Non-Splenomegaly Group (n=261)	*t-value*	*p-value*
Delusions	2.98±1.51	2.95±1.49	0.146	0.884
Conceptual Disorganization	2.01±1.21	2.02±1.18	-0.111	0.912
Hallucinatory Behavior	2.55±1.29	2.45±1.23	0.830	0.407
Excitement	1.98±1.05	1.84±0.96	2.381	0.118
Grandiosity	1.68±1.08	1.68±0.95	0.067	0.947
Suspiciousness/Persecution	2.81±1.38	2.95±1.37	-0.984	0.326
Hostility	1.90±1.14	1.87±1.03	0.276	0.783
P Scale Total Score	15.91±6.70	15.66±6.09	0.397	0.692
Blunted Affect	2.95±1.32	2.69±1.06	2.280	0.023*
Emotional Withdrawal	2.50±1.20	2.41±1.07	0.743	0.458
Affective Communication Disturbance	2.88±1.75	2.34±1.09	3.578	0.000*
Passive-Apathetic Withdrawal	3.19±1.49	2.85±0.98	2.588	0.010*
Difficulty in Abstract Thinking	2.39±1.22	1.96±1.04	3.718	0.000*
Lack of Spontaneity and Flow of Speech	2.48±1.27	2.24±1.22	1.953	0.052
Stereotyped Thinking	2.27±1.14	1.98±1.03	2.703	0.007*
N Scale Total Score	18.67±7.82	16.48±5.37	3.167	0.002*
Poor Grooming and Hygiene	1.87±1.39	1.89±1.29	-0.122	0.903
Anxiety	1.46±1.03	1.95±1.09	-4.708	0.000*
Guilt Feelings	1.31±0.74	1.20±0.51	1.616	0.107
Tension	1.30±0.66	1.68±0.96	-4.871	0.000*
Mannerisms and Posturing	1.28±0.74	1.43±0.85	-1.797	0.073
Depression	1.27±0.69	1.44±0.77	-2.391	0.017*
Retardation	1.42±0.93	1.72±1.05	-3.045	0.002*
Uncooperativeness	1.88±1.23	1.95±1.20	-0.557	0.578
Abnormal Thought Content	2.89±1.53	2.90±1.43	-6.830	0.322
Disorientation	1.25±0.67	1.28±0.72	-0.311	0.756
Poor Impulse Control	1.42±0.81	1.51±0.83	-1.171	0.242
Preoccupation	3.32±1.20	3.35±1.30	-2.632	0.309
Active Social Avoidance	2.42±1.46	2.85±1.33	-3.056	0.002*
Orientation	1.97±1.04	2.02±1.20	-1.949	0.105
Work Inability	1.28±0.88	1.52±0.91	-2.612	0.009*
Lack of Insight	1.25±0.71	1.32±1.26	-3.127	0.320
G Scale Total Score	26.39±12.40	28.81±10.47	-2.658	0.471
PANSS Total Score	59.98±16.86	62.94±19.87	-1.649	0.100

PANSS: Positive and Negative Syndrome Scale; P Scale: Positive Scale; N Scale: Negative Scale; G Scale: General Psychopathology Scale. *$p < 0.05$ indicates statistical significance.
